# Invasive Non-Typhoidal *Salmonella* Typhimurium ST313 Are Not Host-Restricted and Have an Invasive Phenotype in Experimentally Infected Chickens

**DOI:** 10.1371/journal.pntd.0002487

**Published:** 2013-10-10

**Authors:** Bryony N. Parsons, Suzanne Humphrey, Anne Marie Salisbury, Julia Mikoleit, Jay C. D. Hinton, Melita A. Gordon, Paul Wigley

**Affiliations:** 1 ZIPPP Group, Institute of Infection and Global Health, University of Liverpool, Neston, Cheshire, United Kingdom; 2 Institute of Translational Medicine University of Liverpool, Liverpool, United Kingdom; 3 Institute of Integrative Biology, University of Liverpool, Liverpool, United Kingdom; University of Tennessee, United States of America

## Abstract

*Salmonella enterica* serovar Typhimurium Sequence Type (ST) 313 is a major cause of invasive non-Typhoidal salmonellosis in sub-Saharan Africa. No animal reservoir has been identified, and it has been suggested that ST313 is adapted to humans and transmission may occur via person-to-person spread. Here, we show that ST313 cause severe invasive infection in chickens as well as humans. Oral infection of chickens with ST313 isolates D23580 and Q456 resulted in rapid infection of spleen and liver with all birds infected at these sites by 3 days post-infection. In contrast, the well-defined ST19 *S*. Typhimurium isolates F98 and 4/74 were slower to cause invasive disease. Both ST19 and ST313 caused hepatosplenomegaly, and this was most pronounced in the ST313-infected animals. At 3 and 7 days post-infection, colonization of the gastrointestinal tract was lower in birds infected with the ST313 isolates compared with ST19. Histological examination and expression of CXCL chemokines in the ileum showed that both D23580 (ST313) and 4/74 (ST19) strains caused increased CXCL expression at 3 days post-infection, and this was significantly higher in the ileum of D23580 vs 4/74 infected birds. At 7 days post-infection, reduced chemokine expression occurred in the ileum of the D23580 but not 4/74-infected birds. Histological analysis showed that D23580 infection resulted in rapid inflammation and pathology including villous flattening and fusion at 3 days post-infection, and subsequent resolution by 7 days. In contrast, 4/74 induced less inflammation and pathology at 3 days post-infection. The data presented demonstrate that ST313 is capable of causing invasive disease in a non-human host. The rapid invasive nature of infection in the chicken, coupled with lower gastrointestinal colonization, supports the hypothesis that ST313 is a distinct pathovariant of *S.* Typhimurium that has evolved to become a systemic pathogen that can cause disease in several hosts.

## Author Summary

*Salmonella enterica* serovar Typhimurium usually causes foodborne diarrheal infections in humans. Recently variants of *S*. Typhimurium have emerged in Africa associated with invasive and often fatal disease, especially in adults infected by HIV and children with malaria or malnutrition. Analysis of these Sequence Type (ST) 313 isolates has identified significant genome degradation, compared with the ST19 genotype that typically causes diarrhea in humans. This is characterised by pseudogenes and deletions observed in the human-adapted *S*.Typhi. This has led to the hypothesis that the ST313 has become specifically adapted to humans. Here, we show ST313 also cause invasive disease in chickens, a common source of human *Salmonella* infection. As in humans, ST313 strains are more invasive than ST19 *S*. Typhimurium, and also cause less intestinal colonization. However, unlike *Salmonella* serotypes associated with invasive disease in the chicken, ST313 causes inflammation and damage to the intestine. These findings suggest that ST313 is not host-restricted to humans, and may have evolved to cause a more invasive disease in a range of hosts. We highlight the fact that animals, including chickens, could still be a source of ST313 and that further epidemiological research is needed into potential animal reservoirs of ST313 in Africa.

## Introduction

Invasive non-typhoidal salmonellosis (iNTS) has emerged as the most common bloodstream infection in sub-Saharan Africa, frequently as a co-infection with HIV or malaria, leading to mortality events of up to 25% [1]. The predominant *Salmonella* serovar associated with in iNTS is *Salmonella enterica* serovar Typhimurum, though other serovars including *S.* Enteritidis have also been identified [2]. In recent years a highly invasive multi antimicrobial drug resistant (MDR) *S*. Typhimurium variant with a distinct genotype, Sequence Type 313 (ST313), has emerged as a novel pathogenic clade [3]. Although data are sparse, ST313 appear to be less frequently associated with gastroenteritis than ‘classical’ ST19 isolates of *S*. Typhimurium in sub-Saharan Africa. Genomic analysis of D23580, an MDR ST313 isolated in Blantyre, Malawi, revealed that ST313 has undergone genomic degradation including pseudogene formation and chromosomal deletion that is a feature of host-adapted serovars such as *S*. Typhi and *S*. Gallinarum, that cause systemic typhoidal disease in their hosts [4], [5]. Furthermore, epidemiological investigation has been unable to determine an environmental or zoonotic source of iNTS, with transmission being considered likely to be through direct or indirect human-to-human routes and with asymptomatic carriage potentially playing a role [1], [3], [6]. Consequently, it has been suggested that ST313 is restricted to human infection, or at least adapted to a niche of immunosuppressed HIV or malaria-infected patients [1], [3]. However, potential domestic or wild animal sources of infection have been incompletely investigated. Despite this gap in our understanding of the source of ST313, genetic analysis indicates it has probably evolved in the last 30 years and is split into two distinct, but closely related phylogenies, that have evolved sequentially, likely driven by the use of antimicrobials and the emergence of HIV [3].

Although there is currently no epidemiological evidence that birds are a source of ST313 infection, they are perhaps the most likely zoonotic source of *Salmonella*. In Africa, domestic chickens live in close contact with humans in both urban and village communities, often being housed overnight in the family home [7]. Infection of the chicken with pathovars of *S*. Typhimurium like ST19 can persist within the gastrointestinal tract leading to fecal shedding into the environment, contamination of meat and fecal contamination of eggs [8]. Furthermore, *Salmonella* may be carried and transmitted by wild birds, a source that has been associated with major *Salmonella* outbreaks caused by contamination of chocolate and peanut products in both the UK and USA in recent years [9]. Wild birds have not been investigated for carriage of ST313, whilst only a limited number of live chickens and poultry meat have been sampled for iNTS in Africa [6], [10]. This type of survey is complicated because the *Salmonella* carriage rate in healthy wild birds in the UK is around 0.2%, meaning detection of *Salmonella* in wild hosts may require large numbers of animals to be sampled if carriage is at a similar low rate [11]. Intriguingly *S.* Typhimurium of the same phage type, DT56var, is also associated with invasive salmonellosis without gastroenteritis in wild passerine birds such as greenfinches in the UK [11], [12]. This genotype, ST568, is rarely found in human gastroenteritis cases in the UK but has been found in cases of iNTS in sub-Saharan Africa [3].

Experimental infection of the chicken with *S*. Typhimurium has focused primarily on the role of the chicken as a reservoir for human foodborne infection and control of infection in poultry production [8]. Although infection is usually sub-clinical, except in newly hatched chicks, the mechanisms of pathogenesis and host response to infection are broadly similar to those seen in mammalian models of gastroenteritis, with the induction of an inflammatory response largely mediated through the actions of the SPI1 T3SS and host recognition via TLR5 and TLR21 resulting in increased expression of CXC chemokines and an influx of polymorphonuclear cells into the intestine associated with architectural damage [13]–[17]. Unlike mammalian models there is little or no diarrhoea, though colonization of the chicken gastrointestinal tract, and in particular the two large blind ceca that sit at the junction of the ileum and colon, occurs at levels of up to 10^9^ CFU per gram of cecal content [18], [19]. This is usually accompanied by invasion into the spleen and liver that is typically cleared by a T_H_1 dominated immune response around 14–21 days post infection, though gastrointestinal infection may persist for 10 weeks or more [20], [21]. The chicken represents a potentially useful model to study invasive salmonellosis in an immunocompetent host without the need to manipulate the gut microbiota.

In this study we use the chicken as an experimental host to address major questions of the biology of iNTS isolates: Firstly, is *S*. Typhimurium ST313 restricted to humans or is it capable of causing invasive disease and/or colonising a major food animal species? Secondly, does the infection biology of *S*. Typhimurium ST313 differ significantly from that of classical *S*. Typhimurium ST19 in the chicken? Finally, can this animal model potentially inform the basis of fundamental phenotypic differences between the ST19 and ST313 *S*. Typhimurium genotypes? We therefore studied the ability of ST313 to colonize the gut and to invade systemic sites, to elicit expression of key CXC chemokines in the gut and whether, like ST19, ST313 infection leads to an enteropathogenic inflammatory response.

## Materials and Methods

### Ethics statement

All work involving animals was conducted in accordance with UK legislation governing experimental animals under project licences PPL 40/3063 and PPL40/3652 and was approved by the University of Liverpool ethical review process prior to the award of the licences. All animals were checked a minimum of twice daily to ensure their health and welfare.

Bacterial isolates from human subjects were from the University of Liverpool and Malawi-Liverpool-Wellcome Trust (MLW) Centre Blantyre, Malawi joint culture collection. All patient details were anonymous to this study other than isolation from adult or child.

### Bacterial isolates

The MDR *S*. Typhimurium ST313 isolates Q456 and D23580 were isolated from blood of adult (Q456) and paediatric (D23580) patients at Queen Elizabeth Central Hospital, Blantyre, Malawi as previously described and both have been genome sequenced [4], [22]. The *S*. Typhimurium ST19 isolates 4/74 and F98 are well-characterized isolates of defined virulence and colonization in the chicken [15], [23]. All isolates were maintained as frozen stocks at −80°C. Isolates were grown in Luria Bertani broth at 37°C in a shaking incubator at 150 rpm for 18 h prior to use.

### Experimental infections

#### Experiment 1

Forty one day-old unvaccinated female Lohmann Brown chicks were obtained from a commercial hatchery and housed in floor pens as groups of 10 and maintained at a temperature of 30°C. Chicks were given *ad libitum* access to water and a vegetable protein-based pelleted diet (SDS, Witham, Essex, UK). Prior to infection birds were cloacally swabbed to ensure they were *Salmonella*-free. At two weeks of age, individual groups were infected by oral gavage with 10^8^ CFU of one of the *S*. Typhimurium isolates F98, 4/74, D23580 and Q456 in 0.2 ml LB broth. At 3 and 7 days post-infection five birds from each group were killed by neck dislocation. At post mortem analysis gross pathological changes were recorded and the spleen, liver and cecal contents removed aseptically for quantitative bacteriology on modified Brilliant Green Agar as previously described [24], [25]. Samples negative by direct plating were enriched by overnight incubation in selenite broth to determine presence or absence of *Salmonella*.

#### Experiment 2

Thirty female Lohmann Brown chicks were housed in three groups of 10 and housed under the conditions described above. At two weeks of age birds were infected with either *S*. Typhimurium 4/74 or D23580 as described above. One further group was mock-infected with sterile PBS as a control. At 3 and 7 days post-infection 5 birds from each group were killed by neck dislocation. Samples were taken for bacteriology as described above. Samples of liver, spleen and ileum were taken into 4% paraformaldehyde for histology and a sample of ileum and cecal tissue was taken into 0.5 ml RNA*later* stored overnight at 4°C then at −20°C for extraction of RNA to determine expression of key CXC chemokines.

### Expression of CXC chemokines in the intestinal tract

Tissue samples from the ileum and ceca of infected and control animals were collected and stored in 500 µl of RNA*later* (Sigma-Aldrich, Poole, Dorset, UK) at −20°C. Total RNA was isolated from 20–30 mg of tissue using an RNeasy mini kit (Qiagen, West Sussex, UK), according to the manufacturer's instructions. Isolated RNA was eluted into 50 µl RNase-free water and stored at −80°C.

Expression of mRNA for chemokines CXCLi1 and CXCLi2 in these tissues was measured using real-time qRT-PCR. Primer and probe sequences for chicken chemokines and 28S rRNA were used as described previously [26]. 20 µl-reaction mixtures for one-step qRT-PCR were prepared using the RotorGene Probe RT-PCR kit (Qiagen, West Sussex, UK) and contained 1 µl total RNA, 10 µl RotorGene Probe RT-PCR Master Mix, 0.2 µl RotorGene RT Enzyme Mix, 1.6 µl of each primer (at 10 µM), 0.8 µl of probe (at 5 µM) and 4.8 µl of RNase-free water. Triplicate reactions were set up for each sample using an automated QIAgility system (Qiagen, West Sussex, UK). Real-time RT-PCR was performed on a RotorGene Q system (Qiagen, West Sussex, UK) with the following reaction profile: 50°C for 10 min to facilitate reverse transcription of RNA to cDNA, followed by 95°C for 5 min, and 40 repeat cycles of 95°C for 5 s and 60°C for 10 s.

Expression of the target gene was determined using the cycle threshold (C_T_) value relative to that for the 28S rRNA reference gene (ΔC_T_). Results are expressed as fold-changes in corrected target gene expression (ΔC_T_) in infected animals relative to the controls (2^−[ΔΔCT]^).

### Histopathology

Samples of ileum, liver and spleen from birds were fixed in 4% paraformaldehyde for 24–48 h, then trimmed and routinely paraffin wax embedded. Sections (3–5 µm) were prepared and stained with haematoxylin-eosin (HE) and histologically examined to determine any histological changes in response to infection. All sections were examined blind by two individuals. Histopathology was scored individually for each tissue from each animal based on the scoring system detailed in Table 1.

**Table 1:** Histopathology scoring system for experimental avian salmonellosis.

Score	Tissue		
	Ileum	Liver	Spleen
0	Normal	Normal	Normal
1	Small increase in heterophils and macrophage numbers in villi or underlying epithelium. No damage	Small increase in dispersed inflammatory cells	Small increase in dispersed inflammatory cells
2	Increased numbers of heterophils associated with epithelium and lamina propria. Slight thickening/edema of villi.	Increased numbers of inflammatory cells throughout tissue. Small foci of heterophils or macrophages	Increased numbers of inflammatory cells throughout tissue. Increased numbers of monocytic cells in red pulp
3	Extensive inflammatory influx. Thickening and/or flattening of villi.	Formation of small granuloma-like lesions. Extensive influx of inflammatory cells	Extensive influx of inflammatory cells. Increased lymphocyte and monocyte infiltration into white pulp. Some thickening of outer capsule
4	Extensive flattening/blunting of villi. Crypt hyperplasia.	Numerous small or large granuloma-like lesions. Small areas of necrotic damage	Massive cellular infiltration into both white and red pulp. Thickening of splenic capsule
5	Complete loss of villus structure	Large areas of necrotic damage leading to loss of organ structure.	Areas of necrotic damage with some loss of defined red and white pulp structure. Some loss of capsule integrity.

Scoring system based on histopathological descriptions for experimental infection of the chicken with *S*. Typhimurium (WIthanage et al., 2004, 2005) and *S.* Gallinarum (Wigley et al 2002).

### Statistical analysis

Statistical analysis was performed using SPSS version 20.0 software (IBM). Bacterial counts were compared between individual infected groups using a Kruskal-Wallis test as distribution of data was not normal. Comparison of CXCL expression between groups was made using ANOVA. Comparison of pathology scores was made using the Mann-Whitney U test. Significance between the values were taken if *P*<0.05.

## Results

### ST313 *S*. Typhimurium shows a phenotype of rapid invasion in the chicken

**Figure 1:**
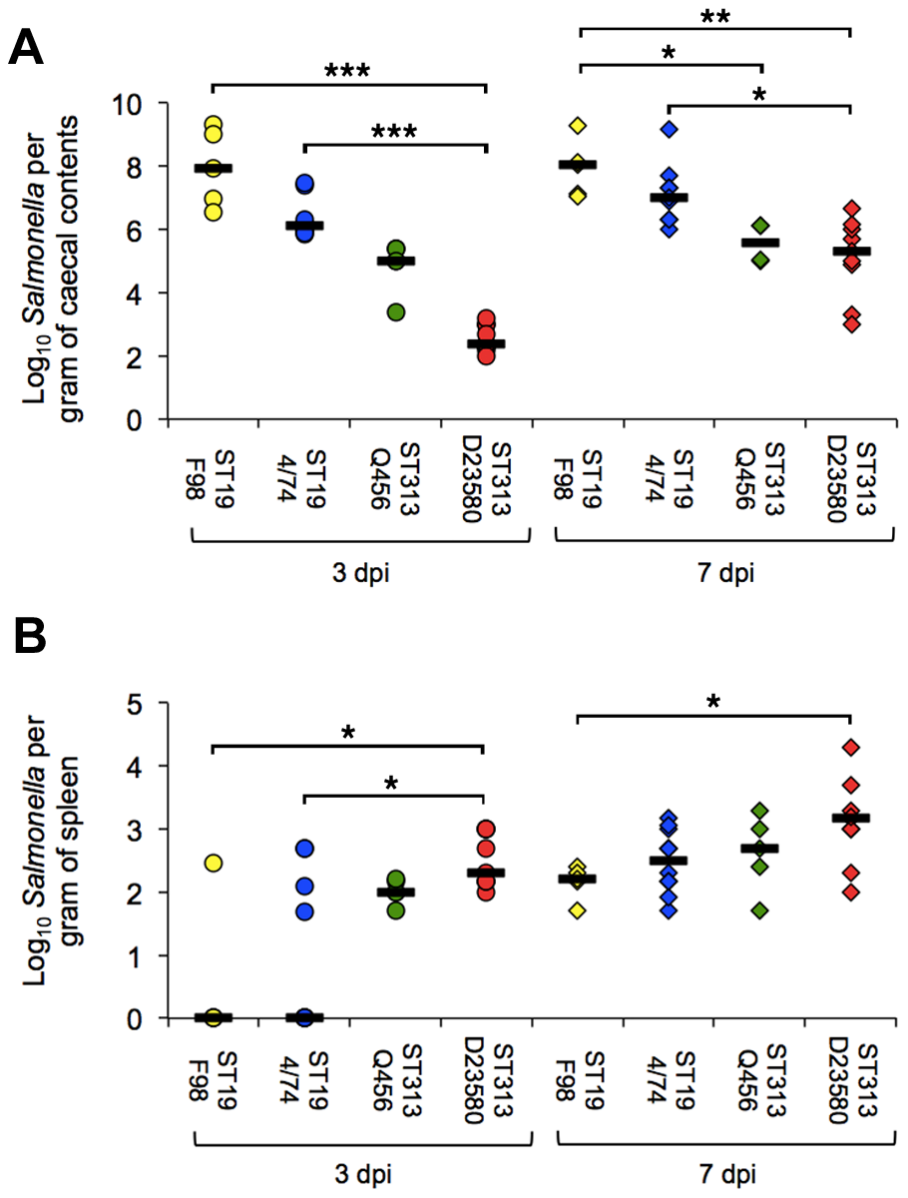
Viable counts of *Salmonella* Typhimurium ST313 and ST19 in cecal contents and spleen following oral infection of the chicken. Viable counts (CFU/g) of cecal content (A) and spleen (B) at 3 and 7 days post-oral infection of two week old commercial egg laying chicks with 10^8^ CFU ST313 (D23580, Q456) and ST19 (F98, 4/74) *S.* Typhimurium. Counts are shown as individual birds with the bar representing the median value. Five birds from each group were killed for sampling at each time point (n = 5) in each experiment. Data shown for D23580 and 4/74 (n = 10 per time point) is combined data for two separately repeated experiments. Statistical comparison was made using a Kruskal-Wallis test. *, P<0.05. **, P<0.01. ***, P<0.001.

Both ST313 isolates were found to colonize the ceca (Figure 1A) and invade the spleen (Figure 1B) and liver (data not shown) at 3 days post-infection with *Salmonella* detected in all birds in these groups. In contrast, *Salmonell*a was detected in the spleen of only one of five birds infected with strain F98, three out of ten birds challenged with 4/74 and not in the liver of any bird. Furthermore, the bacterial counts showed significantly higher infection with D25380 than F98 (P = 0.025) or 4/74 (P = 0.019). By 7 days post-infection S*almonella* was detected in the spleens and livers of all birds, though the splenic counts remained at statistically significantly higher levels in the D23580 infected group in comparison with F98 (P = 0.045) but not 4/74 (P = 0.15).

### ST313 colonizes the intestinal tract but at a lower level than ST19

All ST19 and ST313 isolates colonized the ceca of the chicken by 3 days post-infection and remained colonized at 7 days post-infection (Figure 1A). Both ST313 isolates colonized at significantly lower levels than either F98 (P = 0.033 for Q456, P = 0.002 for D23580 at 7 days post infection) or 4/74 (P = 0.036 for Q456, P = 0.015 for D23580), though both ST19 isolates gave similar levels of colonization. Examination of gross pathology showed rapid onset of moderate hepatosplenomegaly consistent with salmonellosis for both ST313 strains by 3 days post infection, but this was not seen for the ST19 strains. By 7 days post-infection the level of hepatosplenomegaly was similar in all infected birds, and consistent with previous infection studies with both 4/74 and F98 [20]. At 3 days post infection both isolates showed signs of mild inflammation throughout the ileum with mild hyperemia and reddening of the ileal wall. At 7 days post infection no gross inflammation was observed in the ilea of the D23580-infected group, but inflammation was still observed in the 4/74-infected group.

### ST313 infection elicits a rapid intestinal CXC chemokine response that results in intestinal inflammation

**Figure 2:**
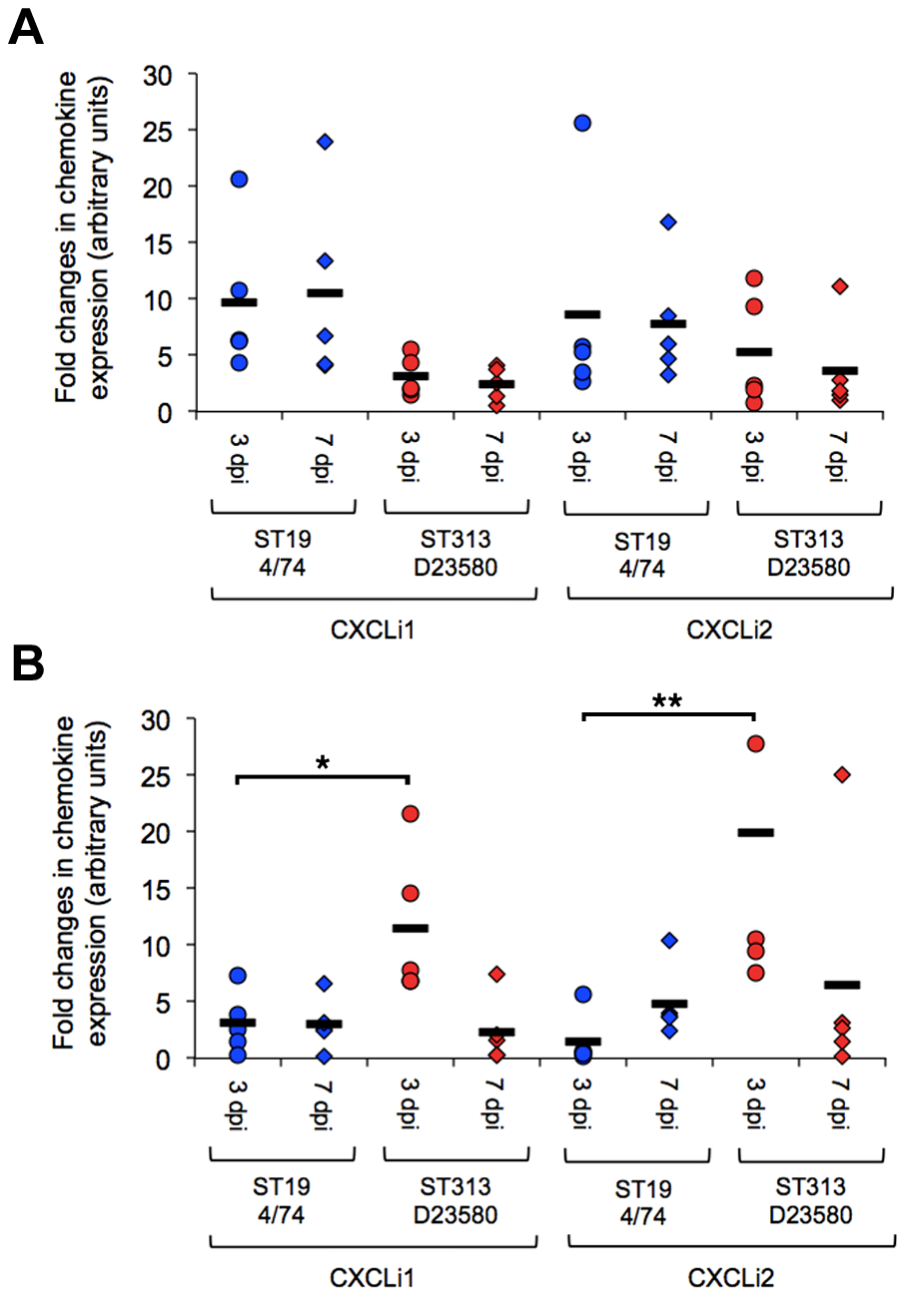
Expression of CXCL chemokines in the chicken gastrointestinal tract following infection with *S*. Typhimurium ST313 or ST19. Expression of the chemokines CXCLi1 and CXCLi2, considered to be orthologous to mammalian IL-8, were determined by qRT-PCR. Relative expression in ceca (Figure 2A) and ileum (Figure 2B) of ST313 (D23580) and ST19 (4/74) infected groups were made in comparison to mock-infected control birds by (2^−[ΔΔCT]^) method. Data is expressed as fold changes in expression in individual birds with the bar representing the mean value. Expression was determined in five birds per group at each time point. Statistical comparison was made by ANOVA. *, P<0.05. **, P<0.01.

**Figure 3:**
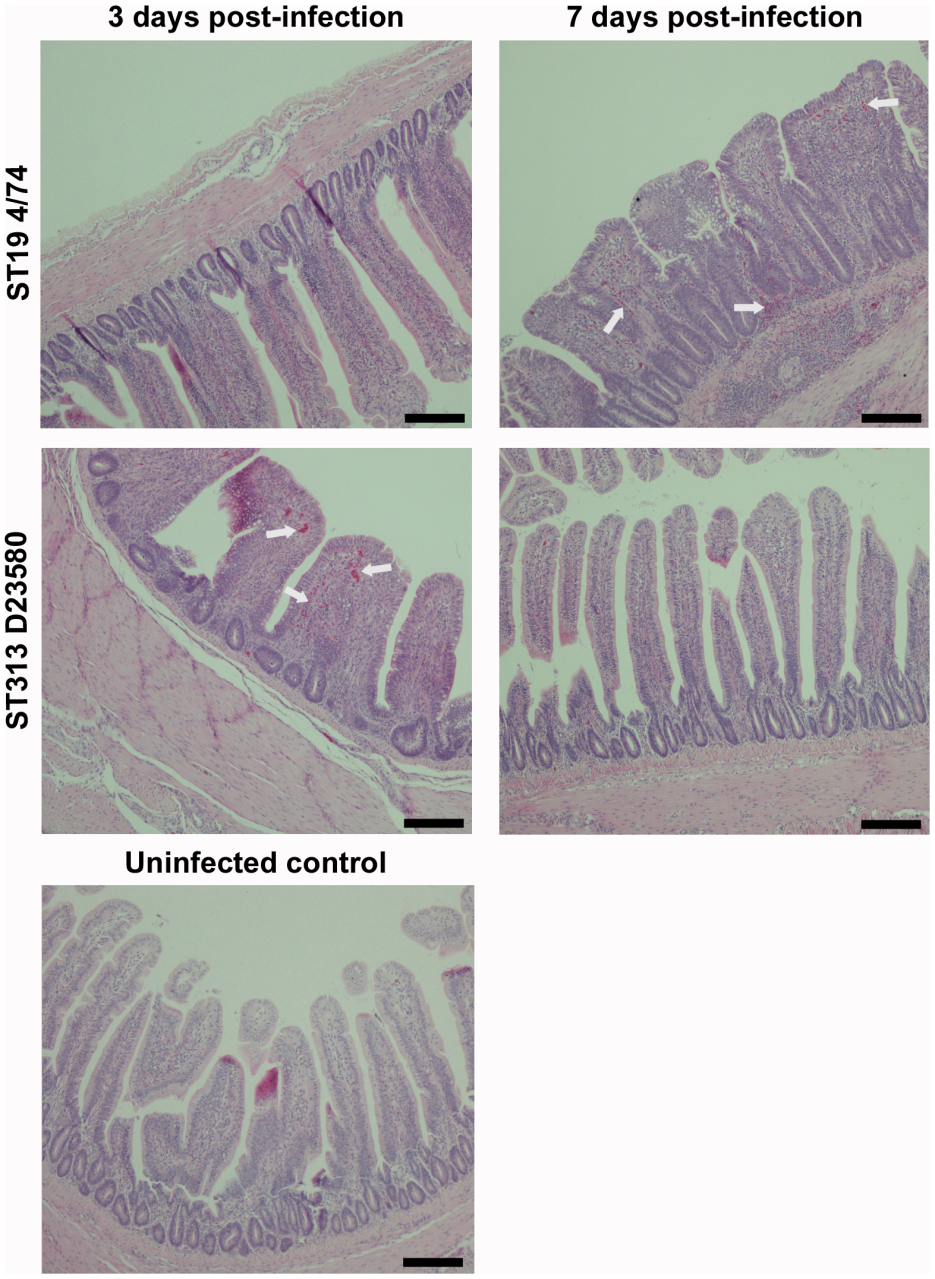
Histopathological changes in the ileum following infection with *S*. Typhimurium ST313 or ST19. Photomicrographs (×400 magnification) of histological changes in H and E stained fixed ileal tissue following infection with *S*. Typhimurium ST313 show a rapid inflammation at three days post-infection leading to villus fusion and flattening accompanied by infiltration of lymphocytes and polymorphonucelar cells (heterophils) and crypt hyperplasia. By seven days post-infection, inflammation and pathology is reduced. In birds infected with ST19 (4/74) *S.* Typhimurium the inflammatory process appears slower with limited inflammation at three days post infection, but substantial damage to the structure of the ileum at seven days post-infection with a high degree of villus damage and large areas of inflammatory infiltration. Arrows indicate heterophil influxes. Scale bar = 100 µm.

Infection with ST313 D23580 elicited a mean 11- fold increase in CXCLi1 gene expression and 20- fold increase CXCLi2 gene expression in the ileum at 3 days post-infection (Figure 2B). *S*. Typhimurium 4/74 also elicited expression of these pro-inflammatory signals at 3 days post-infection in the ileum but at a significantly lower level than D23580 (P = 0.029 for CXCLi1, P = 0.009 for CXLi2). Conversely, 4/74 appeared to elicit higher levels of expression of CXCLi1 and CXCLi2 in the cecal tissue than D23580, but this was not statistically significant for either chemokine (Figure 2A). At 7 days post infection, expression of both CXC chemokines fell in the D23580-infected group, but remained at a similar level in the 4/74-infected group. Expression remained at similar levels in the ceca at 7 days post-infection for both D23580 and 4/74. Histological examination of the ileum as early as day 3 revealed that D23580 infection led to a rapid influx of heterophils to the ileum, leading to flattening and fusion of the villi, consistent with the levels of CXC chemokine expression (Figure 3 and Table 2). At 7 days post-infection, little intestinal inflammation was seen in the D23580 infected group and the inflammation-associated architectural disturbance had largely resolved, whereas the 4/74 infected group continued to show inflammation, flattening of the villi and crypt hyperplasia at significantly higher levels than in the D23580-infected group (P = 0.014) (Table 2). These findings suggest both isolates elicit an inflammatory CXC chemokine response in the intestine and that the resulting inflammation is perhaps slower in onset but more prolonged following infection with the ST19 isolate 4/74. This differential inflammatory response may simply reflect the higher bacterial load in the gut, in keeping with the finding of heavily colonized ceca in birds infected with the ST19 isolate. The increased inflammation observed early in infection with D23580 is consistent with rapid invasion via the ileum as a potential route of entry.

**Table 2:** Mean and median (with range) histopathology scores in spleen, liver and ileum of two-week old chickens experimentally infected with *S*. Typhimurium D23580 (ST313) or 4/74 (ST19).

	3 days post infection				7 days post infection			
Tissue	D23580		4/74		D23580		4/74	
	Mean	Median (Range)	Mean	Median (Range)	Mean	Median (Range)	Mean	Median (Range)
**Spleen**	1.8	2 (1–2)	0.6	1 (0–1)	2.4	2 (2–3)	2.2	2 (1–3)
**Liver**	1.8	2 (1–2)	0.6	1 (0–1)	3.2	3 (2–4)	2.2	2 (2–3)
**Ileum**	2.2	2 (1–3)	2.4	3 (1–3)	0.6	1 (0–1)	2.6	3 (1–4)

Scores based on blind assessment based on pathology score outlined in Table 1 conducted by two individuals. Statistical comparison was made between the two infected groups using Mann Whitney U-test and indicates significantly greater pathology in the spleen and liver (P = 0.016) in the D23580 infected group at 3 days post infection and liver and 7 days post infection (P = 0.032) consistent with systemic salmonellosis associated with an invasive phenotype. Both isolates elicit a moderate inflammatory response in the ileum at 3 days post infection, albeit slightly greater in the 4/74-infected birds. At 7 days post infection this has largely resolved in D23580-infected birds but remains significantly higher in 4/74 infected birds (P = 0.014). n = 5 for each group per time point.

**Figure 4:**
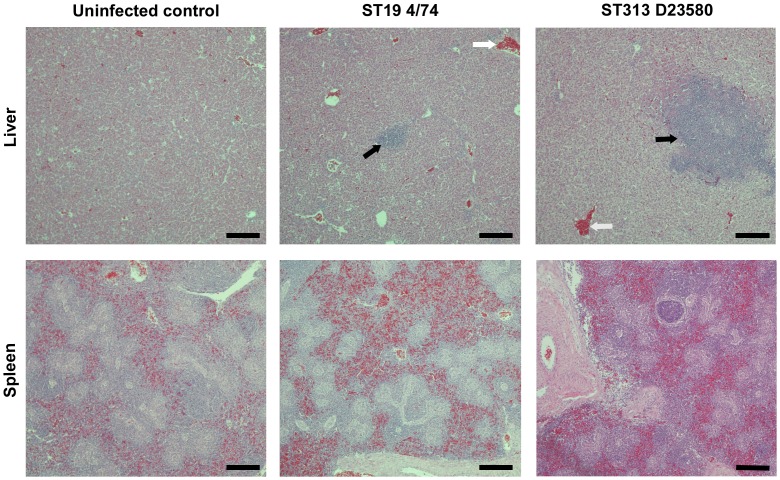
Histopathological changes in liver and spleen following infection with *S*. Typhimurium ST313 or ST19. Photomicrographs (×400 magnification) of histological changes in H and E in fixed splenic and liver sections at seven days post infection. Both ST313 (D23580) and ST19 (4/74) infection result in hepatosplenomegaly as shown by the influx of inflammatory cells, and this is greater in ST313 infection. Infection also results in granuloma-like lesions typical of avian salmonellosis, which are larger and more numerous in the ST313 infected birds. Black arrows indicate granuloma-like lesions and white arrows indicate heterophil influxes. Scale bar = 100 µm.

Hepatosplenomegaly was observed following infection at 7 days post infection with both isolates in comparison with the uninfected controls, but only in the D23580-infected group at 3 days post infection. Histology revealed some inflammation in the liver and spleen following infection with both isolates (Table 2), but this was significantly greater in the D23580 infected group (P = 0.032), consistent with more rapid systemic infection. By 7 days post infection, both isolates caused leukocytic influxes into the spleen and liver (Figure 4) and formed granuloma-like lesions consistent with previous descriptions [20]. However, the degree of inflammation remained significantly greater in the ST313 infected livers than 4/74 at day 7 (P = 0.016), and lesions were both larger and more numerous in livers of the ST313-infected group. Differences in the spleen at 7 days post infection were less pronounced, though pathological changes during avian systemic salmonellosis are frequently more apparent in the liver [27]

## Discussion

It has been proposed that *S*. Typhimurium ST313 are adapted to human infection [1], [3] and might be host-restricted. Here we clearly demonstrate that ST313 *S*. Typhimurium are not restricted to humans and that an invasive phenotype may occur in other host animals. The data presented here indicate that the *S*. Typhimurium genotype ST313 is capable of causing invasive infection in the chicken, accompanied by intestinal inflammation and colonization of the chicken gastrointestinal tract. The infection phenotype differs from ‘classical’ ST19 *S*. Typhimurium in that systemic invasion appears to be more rapid and, in D23580 at least, occurs at a higher level than in either ST19 isolates tested. The data suggest that the site of rapid invasion and the associated early inflammation is largely localized to the ileum, though additional studies would be needed to confirm this. Although cecal colonization of the gastrointestinal tract by ST313 is significantly lower than the ST19 isolates examined, ST313 clearly retains the ability to colonize the ceca. The reasons for the lower levels of colonization are not clear; we did observe a trend for lower levels of chemokine expression in ceca, but this was not statistically significant, and may simply reflect the presence of lower number of organisms. Given that ST313 are less frequently associated with human gastrointestinal infection than ST19 (although they may cause a proportion of diarrheal disease in Africa [28]) it is tempting to consider ST313 to be evolving towards a more ‘systemic lifestyle’ because they most commonly cause bloodstream infections in Africa. Although the kinetics of ST313 infection of chickens appear to be more rapid than either *S*. Typhimurium 4/74 or F98, the underlying pathogenesis appears similar. D23580 elicits a rapid inflammatory response in the ileum with expression of CXC chemokines and an early influx of heterophils, the avian equivalent of the neutrophil. This is accompanied by inflammation and a leukocytic influx into the spleen and liver leading to hepatosplenomegaly. What differs is that this response is more rapid and intense than following infection with the ST19 isolate 4/74. Infection is, interestingly, therefore more akin to the pathology found following infection with ‘classical’ ST19 isolates rather than the ‘stealth infection’ found with the host-restricted *S*. Gallinarum and *S*. Pullorum that do not elicit any inflammatory response in the gut [29]. Furthermore, our data show that ST313 can establish both systemic and gastrointestinal infection in the chicken in clear contrast to the fully human-adapted *S*. Typhi, which is unable to establish infection in chicks or embryos (Stanley Falkow, personal communication). These findings mirror the situation in humans, where ST313 forms a distinct invasive pathotype of *S*. Typhimurium that primarily manifests in immunosuppressed patients and can elicit inflammatory enteropathogenic responses that potentially may cause gastroenteritis in healthy individuals [28]. The close association between ST313 and invasive disease in humans may be a consequence of this invasive phenotype coupled to immunological dysfunction as a result of HIV infection [30] or as a consequence of the host response to malaria favoring establishment of systemic salmonellosis [31]. The chicken may also represent a valuable model to further elucidate the behavior of this pathotype in the gut.

Previous studies have failed to determine an animal or environmental reservoir for iNTS in Africa [6], [10]. However, these studies have been limited in size and scope, so the absence of a known wild or domestic animal host is perhaps a consequence of these limitations. ST313 can colonize the intestine of the chicken, albeit not as successfully as other common *S*. Typhimurium pathovars, but sufficiently well to have the potential to be a reservoir of infection. Whilst ST313 is also more invasive in the chicken, the pathology seen is mild compared to that of avian adapted serovars [27]. It is likely that ST313 has an original zoonotic source, but possible that once it entered a susceptible population then person-to-person transmission became more important. Alternatively, it is possible that birds are the natural host of ST313 and that direct transmission from infected feces or via contaminated food may remain an important source of infection. It is difficult to conduct such studies in developing countries such as Malawi, particularly if the presence of a specific pathotype is found relatively infrequently in an animal host. Surveillance for foodborne pathogens such as *Salmonella* is commonplace or indeed a legal requirement in the large commercial poultry industries in North America and Europe [8], but the low intensity backyard production of chickens in countries like Malawi make this considerably more difficult. Furthermore, it is likely to be a challenge to establish whether ST313 has a wild animal source in Africa. The infection of farm animals in the UK is associated with low frequencies of carriage of *Salmonella*, and zoonotic transmission was shown to be an infrequent and stochastic event [11], [12]. Nevertheless, our data show that there is potential for carriage of ST313 in the avian intestines and as a consequence zoonotic transmission. Further understanding of these potential transmission routes is now an important priority.

In summary, the data we present here show that *S*. Typhimurium ST313 are invasive in an animal infection model. Although ST313 isolates show a reduced ability to thrive in the intestinal tract, the level of colonization is sufficient for chicken to act as a potential source of zoonotic infection. Although genomic degradation and the absence of epidemiological evidence of a zoonotic source have led to speculation that the ST313 genotype may be adapted or even restricted to a human host, the data presented here indicate that ST313 are capable of both causing invasive disease and colonizing the intestinal tract of chicken. The rapidly invasive nature of infection in the chicken suggests that ST313 may be a distinct pathotype adapted to cause a invasive salmonellosis, as has been well-characterised in susceptible human hosts.
